# Autonomic Characteristics of Sudden Unexpected Death in Epilepsy in Children—A Systematic Review of Studies and Their Relevance to the Management of Epilepsy in Rett Syndrome

**DOI:** 10.3389/fneur.2020.632510

**Published:** 2021-02-05

**Authors:** Jatinder Singh, Evamaria Lanzarini, Paramala Santosh

**Affiliations:** ^1^Department of Child and Adolescent Psychiatry, Institute of Psychiatry, Psychology and Neuroscience, King's College London, London, United Kingdom; ^2^Centre for Interventional Paediatric Psychopharmacology and Rare Diseases, South London and Maudsley NHS Foundation Trust, London, United Kingdom; ^3^Centre for Personalised Medicine in Rett Syndrome, Institute of Psychiatry, Psychology and Neuroscience, King's College London, London, United Kingdom; ^4^Child and Adolescent Neuropsychiatry Unit, Infermi Hospital, Rimini, Italy

**Keywords:** sudden unexpected death in epilepsy, epilepsy, autonomic dysregulation, Rett Syndrome, pediatric

## Abstract

**Aim:** To systematically identify and critically appraise studies that investigate the autonomic characteristics of Sudden Unexpected Death in Epilepsy (SUDEP) in the pediatric population. We also wanted to explore how this information would be relevant to the management of epilepsy in patients with Rett Syndrome.

**Method:** Using PRISMA guidelines, a systematic review of PubMed, Scopus, Cochrane, PsycINFO, Embase, and Web of Science databases was performed to identify eligible studies. After extracting data from the included studies, a thematic analysis was undertaken to identify emerging themes. A quality appraisal was also done to assess the quality of the included studies.

**Results:** The systematic search revealed 41 records, and 15 full-text articles on the autonomic characteristics of SUDEP in children were included in the final analysis. Following thematic analysis, three themes were identified (I) modulation in sympathovagal tone, (II) pre- and post-ictal autonomic changes, and (III) other markers of autonomic dysregulation in children with epilepsy. Modulation in sympathovagal tone emerged as the theme with the highest frequency followed by pre- and post-ictal autonomic changes. While the themes provide additional insight into the management of epilepsy in the Rett Syndrome population, the quality of evidence concerning the autonomic characteristics of SUDEP in the pediatric population was low and underscores the importance of much needed research in this area.

**Conclusion:** The mechanism of SUDEP in the pediatric population is complex and involves an interplay between several components of the autonomic nervous system. While direct clinical inferences regarding pediatric SUDEP could not be made, the thematic analysis does suggest that in vulnerable populations such as Rett Syndrome, where there is already a pervasive autonomic dysregulation, pro-active surveillance of the autonomic profile in this patient group would be useful to better manage epilepsy and reduce the SUDEP risk.

## Introduction

The management of epilepsy in children is clinically challenging and longitudinal follow-up studies have shown that death associated in young children with epilepsy is greater than the general population (1, 2). When a death occurs suddenly or is unexpected in children with epilepsy the term “Sudden Unexpected Death in Epilepsy” (SUDEP) has been ascribed. A more recent classification has expanded this definition (3) and encompasses the definitions of SUDEP + and takes into account if a comorbid disorder co-exists such as prolonged QT, however, the diagnosis of pure SUDEP is one that is based on exclusion. This disorder also shares substantial overlap with other disorders of sudden death, such as Sudden Infant Death Syndrome (SIDS) and Sudden Unexplained Death in Childhood (SUDC). Some overlapping features include evidence of hippocampal abnormalities, and association with disordered serotoninergic pathways. However, when SUDEP is compared to SIDS and SUDC, the defining feature is the clinical history of epilepsy and the event can occur at any age (4). Even though SUDEP is not dependent on age, some evidence suggests that the risk of SUDEP is about seven times greater in individuals with epilepsy age of onset between 0 and 15 years when compared to an age of onset ≥45 years (5, 6). Others have suggested that in those with childhood onset of epilepsy that does not fully subside, the lifetime SUDEP risk is 8% by 70 years of age (4, 7).

The event of SUDEP is probabilistic, and this is in part seen in the lack of consensus regarding the prevalence rates. While previous guidelines indicate an average incidence of 1.2/1,000 person-years for adults, and 0.2/1,000 person-years for children (8), some others have suggested that the incidence is far higher (9). An estimate of SUDEP incidence of 1.11/1,000 for children has been suggested (10). Further evidence has shown that even when adjusting for comorbid disorders, the risk of sudden death remains high in children with epilepsy (11) and underscores the importance for increased vigilance in this population. In the United Kingdom, deaths from epilepsy are increasing (12) and recent evidence from the North American SUDEP Registry has indicated that SUDEP can occur even in epilepsy that is relatively benign and treatment responsive (13). Despite this awareness, knowledge of SUDEP in the pediatric literature is relatively scarce, especially in neurodevelopment disorders of childhood that present with a clinical history of epilepsy.

Rett Syndrome (RTT) is a complex pediatric neurodevelopmental disorder characterized by comorbid symptoms and developmental delay. The frequency of epilepsy is varied in RTT. Data from the RTT Natural History study suggest a seizure prevalence ranging from 30 to 44%; however, the lifetime prevalence was up to 90% (14). Others have suggested prevalence rates of 82% (15), 76% (16), and 68.1% (17). Anti-seizure medications (ASMs) are also frequently used for treating epilepsy in RTT. In one study, 64% of patients were taking ASMs, and about 17% were reported not having seizures (18). Similarly, the age of the onset of epilepsy in RTT is variable ranging from 1 to 16 years age of onset (mean 5 years) (15). Other data suggest seizure frequency was about 11% in those under 4 years of age to a peak incidence of about 50% in the 16 to <20-year age group (14). Further, only about 8% of patients had onset after 20 years of age (14). In a study of 1,248 patients, the mean onset age of epilepsy was 4.68 ± 3.5 years of age (17). Despite these observations, there is no information in the literature concerning SUDEP in patients with RTT, especially in children. We do not know whether the trajectory of SUDEP changes over the periods of neurodevelopment in RTT, and neither do we know if tracking these changes would help in detecting early epileptic events that might lead to SUDEP.

Patients with RTT are at more risk of sudden death (19) and we know that the underlying epileptic seizures could potentiate brainstem vulnerability thereby increasing the risk of SUDEP in this patient group (20) especially in those with severe cardio-respiratory dysfunction. However, there are additional risk factors regarding SUDEP that should also be considered in the context of RTT. First, evidence (21) has shown that having three or more generalized tonic-clonic (GTC) seizures per year seems to be the highest weighted risk factor for SUDEP (9), followed by ≥13 of any type of seizure in the last 12 months (22). Second, polypharmacy is also an important risk factor, and data has shown that the SUDEP risk is increased in individuals taking ≥3 ASMs compared to monotherapy (22). Third, developmental delay is also suggested to be a risk factor (9, 23, 24). Fourth, children with complex epilepsy especially in those with associated neurodisability might also have an increased SUDEP risk (6, 9). These factors also feature on the SUDEP-7 risk inventory (9). When viewed together, these elements are also transferable risk factors for patients with RTT because this patient group has generalized seizures as a common seizure phenotype, have developmental delay and are usually prescribed with one or more ASM.

Given that the underlying autonomic impairment in RTT could help in identifying events that could lead to SUDEP (20), it would be prudent to explore studies relating to the autonomic profile of children with SUDEP to see if we can identify patterns or hallmark features that might help to detect early changes that lead to SUDEP in RTT. The purpose of this study was (I) to systematically identify studies on the autonomic characteristics of SUDEP in children, (II) to appraise the identified studies to see whether we can recognize profiles or hallmark features of SUDEP in children, and (III) to use this knowledge to develop and propose any intervention that might enable the early detection of events that increase the risk of SUDEP in children with RTT. As the inherent nature of SUDEP is heterogenous, we were especially interested in whether the information extracted from the studies in children could aid in the development of biomarkers that would help to profile RTT patients deemed most at risk i.e., those on multiple ASMs, have a more severe breathing phenotype and have frequent seizures.

## Methods

To perform the systematic review, two authors (JS and EL) independently followed the PRISMA guidelines (25) to search the PubMed, Scopus, Cochrane, PsycINFO, Embase, and Web of Science databases during October 2020 in a blinded manner. To ensure the search was expansive and captured the relevant search terms, the truncation symbol (^*^) was used.

### Search Terms

The following search terms were used:

(Sudden Unexpected Death in Epilepsy OR SUDEP) AND (autonomic variables OR autonomic parameters) AND (child^*^ OR pediatric).

### Population Characteristics

Databases were searched for records that looked for studies in children that mentioned SUDEP.

### Intervention

All studies that mentioned or reported autonomic characteristics or parameters were included.

### Eligibility Criteria

The following inclusion and exclusion criteria were used:

Inclusion Criteria

➢ Full-text records in peer-reviewed academic/scientific journals
➢ Studies or investigations done in humans and available electronically

Exclusion Criteria

➢ Studies not available electronically and not available in English
➢ Reviews, case reports, and preprints.

### Critical Appraisal of Eligible Articles

The quality of the eligible articles was determined using the appraisal checklist developed previously (26) and has been used in systematic reviews of RTT syndrome (20, 27). In the present study, the procedure to critically appraise the 15 articles against the 11 criteria was followed as described in our previous evidence synthesis (20).

### Data Extraction and Analyses

The methods of data extraction and analysis was performed as previously described (20). To minimize bias in the search process, data extraction and analysis, we used the following strategies:

(I) Two authors (JS and EL) blindly and independently performed the systematic review. The eligible articles were based on a consensus agreement between JS and EL. If agreement could not be reached, then the senior author (PS) was consulted.
(II) The first author (JS) performed the manual coding as previously described (20) to identify preliminary themes. The second author (EL) then independently reviewed these themes and then afterwards, a consensus was reached between JS and EL on the themes that emerged. Lastly, the themes were reviewed by the senior author (PS), and the final themes were based on an agreement between all three authors.

The frequencies of the themes were presented using Microsoft Excel software 2016.

## Results

**Figure 1:**
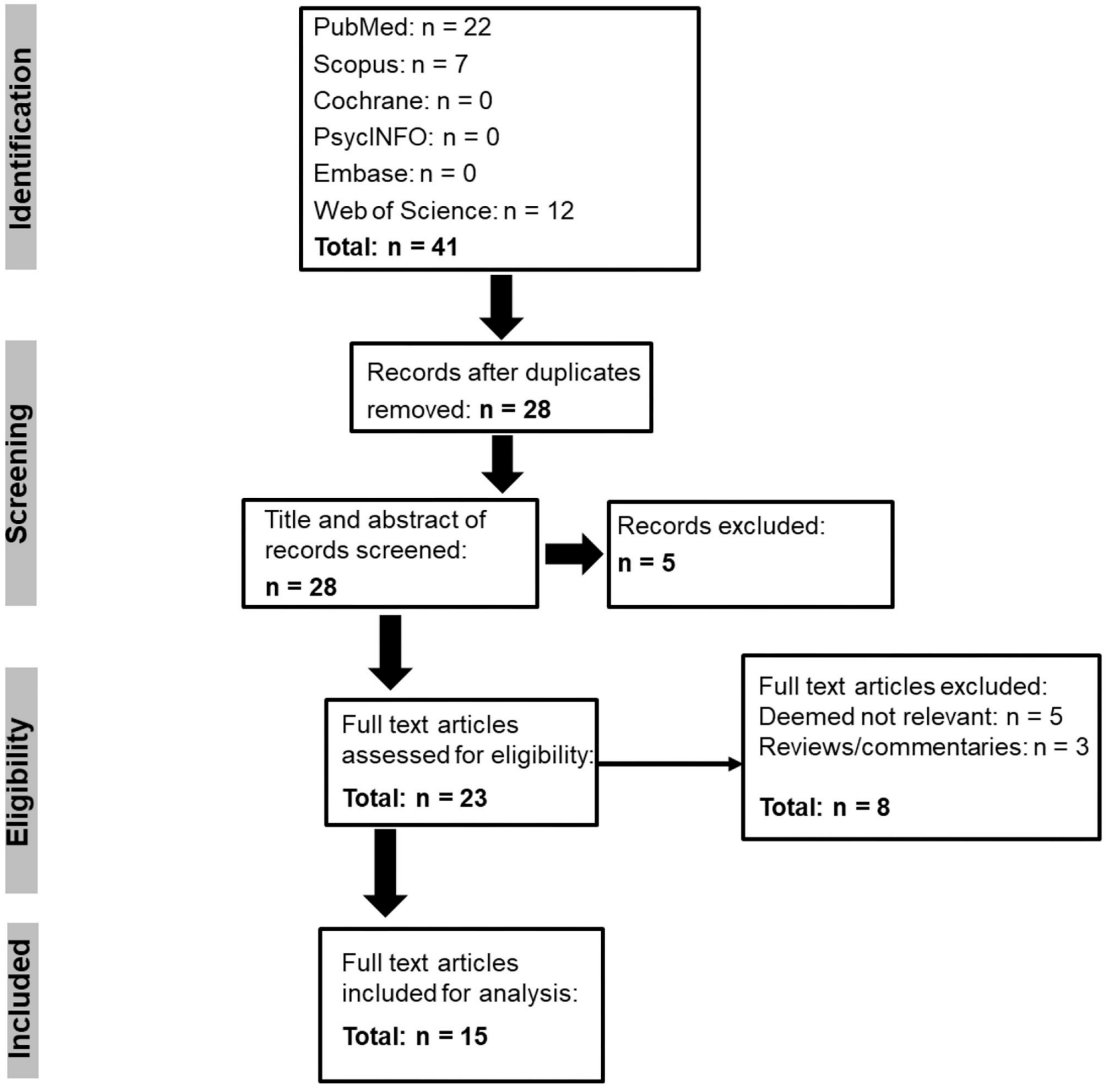
PRISMA flow-diagram.

The systematic search of the databases revealed 41 records, and after duplicates were removed 28 articles remained (Figure 1). The title and abstract of these articles were screened, and five articles were excluded. Twenty three full-text articles were then assessed against the eligibility criteria, and a further eight articles were removed. The remaining 15 full text articles were included in the analysis, and the autonomic characteristics from each of these articles are presented in Table 1.

**Table 1:** Summary of eligible studies relating to the autonomic characteristics of SUDEP.

**Source**	**Demographics**	**Clinical characteristics**	**Assessment methods**	**Relevant autonomic information**
Okanari et al. (28)	• Thirty five children aged between 3 and 18 years who had GCS. • Seventeen age-matched controls.	• In the 35 children, 74 instances of GCS were identified. • Of the 74 GCS, 36 of these also showed PGES and 38 GCS were without PGES.	• Video EEG and ECG (1 lead monitoring). • Pre-, inter-, and post-ictal measurements of HRV parameters including LF, HF, LF/HF, and RMSSD.	• The pre-ictal autonomic parameters LF and HF in children with 36 GCS+PGES was significantly greater (*P* < 0.01) when compared to 38 GCS without PGES. • Post-ictal RMSSD was higher in the GCS+PGES group than the GCS-PGES group (*P* < 0.01) and the pre to post-ictal change in RMSSD was lower in children with GCS and PGES than those that had GCS without PGES (*P* = 0.035). • No changes in inter-ictal HRV parameters were observed among the GCS and the control group. • Measurement of HRV parameters could be useful to identify those subsets of high-risk children such as those with abnormal GCS+PGES changes that might lead to SUDEP.
Pernice et al. (30)	Thirty seven children (*n* = 20 males and *n* = 17 females) aged 6.27 ± 5.1 years of age.	• The children had either focal (*n* = 23) or generalized (*n* = 14) seizures. • Patients were treated with ASMs based on their diagnosis.	Nine HRV parameters were measured in time, frequency, and entropy domains.	• HRV analysis was able to discriminate between focal and generalized seizures. • During the post-ictal phase, children with focal seizures had elevated heart rate, depressed HRV and increases in LF and the LF/HF ratio. • In comparison to children with focal seizures, seizures in children with generalized epilepsy were characterized by increases in the normalized LF, LF/HF ratio, and a lower mean RR interval and RMSDD before the seizure. • Monitoring of HRV can be useful in identifying shifts in the sympathovagal balance reflected by changes in focal seizures or during periods of generalized seizures. • A dominant sympathetic profile and vagal withdrawal are thought to be characteristic in children with generalized seizures during the pre-ictal period.
Yang et al. (32)	• Fifty one patients (*n* = 34 males and *n* = 17 females) aged between 6 and 38 years of age. • Fifty age and gender matched controls.	• All patients had refractory epilepsy and were treated with either one or more ASMs. • The mean (SD) number of seizures per month were 103.1 (174.4). • Of the 51 patients, 38 were on polytherapy and 13 were on monotherapy.	Time, frequency and non-linear domain HRV parameters using 24-h ECG	• The findings showed that patients with refractory epilepsy had significantly lower time, frequency, and non-linear domain parameters than healthy controls. • The difference in the HRV parameters between the epilepsy and control groups was the highest in the early morning. • Altered sympathovagal imbalance as reflected by impaired HRV parameters might be useful for the development of prognostic markers of SUDEP
Sivakumar et al. (35)	• The generalized epilepsy group consisted of 91 subjects with a mean (SD) age: 10.5 (5.0) years. • The comparator group was a control group of 25 subjects with a mean (SD) age: 7.5 (6.4) years	• All subjects had a diagnosis of epilepsy. • Subjects were on a ketogenic diet and were taking ASMs. • During the overnight period, subjects were asked not to take their medications.	• Retrospective review of medical records. • Measurement of HRV, ECG and EEG waveforms. • ECG traces were explored during 30 min of stage 2 sleep.	• In the absence of seizures, there was increased RSA and lower heart rate in children with epilepsy during sleep. • These findings suggest an increased vagal tone in children with generalized seizures. • It was proposed that an increase in parasympathetic tone could precede the onset of epilepsy in children.
Sarkis et al. (36)	• Twenty patients were included. • Seven were in the age range of 11–17 years (younger age group) and 13 were adult patients (18–67 years).	• All patients that had analyses wore an EDA sensor. • MRI lesions were noted in some of the patients. • Mean (range) duration of epilepsy was 11.1 years (1–51) • Focal seizures accounted for 80% of the epilepsy type. • Number (mean [range]) of ASMs was 2.5 (1–5)	• Use of an EDA wrist sensor. • EEG and ECG measurements.	• The study showed that there was a strong correlation between the duration of PGES and age (*P* = 0.004). • When the first GTC seizure was compared between the adult and the younger age groups, it showed that younger patients had a higher EDA amplitude than the adult group (11.80 μS ± 6.94 vs.5.19 ± 3.40 μS *P* = 0.03), suggesting a greater degree of sympathetic activation. • The mean % change in HF power was also higher in the younger age group in comparison to adults (−97.78 ± 6.0 vs.−72.7 ± 15.0, *P* = 0.0016) reflecting increased vagal suppression. • Following a GTC seizure and controlling for PGES duration, patients of a younger age are suggested to have enhanced sympathetic activation and vagal suppression.
Kolsal et al. (37)	• Group 1 (refractory epilepsy; *n* = 20): Mean age ± SD 9.55 years ± 5.02 • Group 2 (controlled epilepsy; *n* = 20): Mean age ± SD 10.1 years ± 4.18 • Group 3 (healthy controls; *n* = 20): Mean age ± SD 10.35 years ± 4.39	• Children with refractory epilepsy were using three or more ASMs. • All patients were assessed by a Pediatric Cardiologist.	• HRV measurements using Holter and 12-lead ECG. • Video EEG • Brain MRI evaluation	• Children with epilepsy have abnormal QTcD and have depressed HRV. • The time domain autonomic parameters RMSSD and SDNN in patients with treatment resistant epilepsy was also lower than the other two groups. • A disruption in the vagal tone reflected by changes in the LF/HF ratio before and during seizures suggests that the sympathovagal balance is considerably stressed in children with epilepsy, and the sympathetic component is thought to dominate before seizure onset.
Jansen et al. (38)	• Seizures were monitored from patients aged 9.2 years • Patients were selected from a group of 35 patients	• EEGs of 80 seizures were analyzed pre- and post- seizure onset. • Seizures were of focal onset (*n* = 40) and of generalized onset (*n* = 40)	• Video EEG • HRV time and frequency domain parameters.	• The R-R interval was useful in detecting pre-ictal heart rate changes in 70% of focal seizures. • In focal seizures, the pattern of mean R-R was different before the seizure onset when compared to after seizure onset, and the duration of pre-ictal HRV to seizure onset is short. • It was proposed that change in heart rate might be useful in detecting aberrant changes that manifest prior to the onset of temporal and frontal lobe seizures in children.
Moseley et al. (29)	• Thirty seven patients (male *n* = 13; female *n* = 24) • Age at admission was: 10.2 ± 4.8 years. • Age at seizure onset was 5.3 ± 4.5 years	• Children were included if they had one documented focal or primary/secondary GTC seizure. • In 40.5% (15) children there was developmental delay.	• EEG alongside ECG and pulse oximetry measurements. • SUDEP-7 inventory score.	• Only GTC seizures were characterized with PGES. • PGES was shown to account for about 16% (27/168) of the seizures in 32% (12/37) of children, and was significantly associated with peri-ictal tachycardia (*P* = 0.019) and hypoxemia (*P* = 0.005). • Mean duration of PGES was 35.1 ± 19.6 s, and in 10 children the PGES was deemed to be prolonged (≥30 s). • Children with PGES also had higher SUDEP-7 inventory scores than children without PGES (4.2 ± 1.3 vs. 2.8 ± 1.4, *P* = 0.007). This might suggest that children with PGES during a GTC seizure could be at higher risk of SUDEP.
Brotherstone and McLellan (39)	• Eleven patients were included with an age range of 3 years 1 month to 60 years 3 months • Six patients were adults (male *n* = 3; female *n* = 3) and five patients were pediatric (male *n* = 4; female *n* = 1).	• From the 11 patients 33 sub-clinical seizures were recorded with a mean duration of 191.1 s ± 136.4 (range: 63–340 s). • The 33 seizures were classified as being generalized (*n* = 19), right temporal lobe (*n* = 9), and left temporal lobe (*n* = 5)	• Prospective measurement of video EEG, ECG, and oxygen saturation recordings. • NeuroScope analysis	• Generalized sub-clinical seizures showed larger increases in cardiac vagal tone and less change in HRV when compared to temporal lobe sub-clinical seizures. • The findings showed that during generalized sub-clinical seizures there is an elevated parasympathetic activity, however, seizures originating from the temporal lobe showed lower parasympathetic activity. • During sub-clinical generalized seizures there is autonomic dysregulation characterized by changes in the parasympathetic component.
Mukherjee et al. (31)	• Group 1 (intractable epilepsy; *n* = 31 [male *n* = 22; female *n* = 9]): Age 22.11 ± 10.18 • Group 2 (well-controlled epilepsy; *n* = 30 [male *n* = 18; female *n* = 12]): Age 19.13 ± 8.72	• All patients were confirmed as having intractable or well-controlled epilepsy. • In Group 1, 25 subjects were on two ASMs while five were treated with three ASMs. • In Group 2, 26 subjects were on one ASM, relatively stable and four were on two ASMs.	• Range of tests for autonomic function including the deep breathing test, Valsalva maneuver, hand grip test, cold pressor test, and head up-tilt test. • Cardiovascular tone (respiration and ECG waveform, and time domain analyses). • Neuropsychological assessment of anxiety using clinician rated questionnaires. • Autonomic symptom score consisting of seven autonomic indices.	• Patients with intractable epilepsy (Group 1) had higher LF and lower HF values than the well-controlled group. • Group 1 was noted to have a higher autonomic dysregulation as evidenced by a higher sympathetic tone, lower parasympathetic tone, and lower parasympathetic reactivity. • It was indicated that patients with intractable epilepsy have a different and more severe autonomic profile than those with well-managed epilepsy, and that these patients could be at higher risk from SUDEP.
Hallioglu et al. (40)	• Group 1 (epilepsy patients on treatment; *n* = 78): Mean age ± SD 7.2 years ± 4.3 • Group 2 (epilepsy patients without treatment; *n* = 14): Mean age ± SD 8.2 years ± 2.7 • Group 3 (healthy controls; *n* = 83): Mean age ± SD 8.1 years ± 3.4	• Of the 92 patients with epilepsy, 14 had a new diagnosis and did not receive ASM. • Of the 78 patients using ASMs 33 used valproic acid. 19 used oxcarbazepine, 11 phenobarbital, 10 were on combined treatments and 5 received other drugs.	• ECGs • Measurement of time and frequency domain HRV indices.	• The findings showed that the HRV and time domain measures (RMSSD, SDNN and HRV triangular index) were decreased in epilepsy patient regardless whether they were on ASM. • However, patients not on any ASMs were said to have a lower parasympathetic activity as indicated by lower HF values and an increased LF/HF ratio. • The parasympathetic autonomic profile is more suppressed in patients not on ASM.
Harnod et al. (41)	• Thirty children (15 males and 15 females) with a mean age 10.9 ± 0.6 years • The control group had 30 individuals (15 males and 15 females) with a mean age of 10.6 ± 0.6 years	• All children with epilepsy had recurrent seizures and were on ASMs • Duration of epilepsy was 6.1 years ± 0.7	ECGs to characterize and assess frequency domain analysis of HRV	• The epilepsy group had lower frequency domain indices (R-R, LF, and HF) when compared to the control group. • It was proposed that in children with intractable epilepsy there is lower HRV due to a decrease in the parasympathetic component.
El-Sayed et al. (42)	• Twenty-five young people (13 males and 12 females) with a mean age 10.36 years ± 4.0 • The control group consisted of 50 individuals (26 males and 24 females) with a mean age of 11.0 years ± 3.5	• Patients had both partial and generalized epilepsy. • Generalized seizures were present in 10 patients and 15 had focal related epilepsy. • Patients were on monotherapy (13 on valproate or 12 on carbamazepine).	• Clinical scoring of five autonomic function test including resting heart rate, heart rate response to deep breathing, Valsalva maneuver, 30:15 ratio heart rate response to standing and blood pressure response to standing. • Time domain HRV measurements.	• SDNN was found to be lower across all age groups. • All patients with uncontrolled epilepsy had abnormal autonomic dysregulation (83% had moderate autonomic and one [17%] had mild autonomic dysregulation). • Seizure type and type of ASM had no discernable effect on the outcome of clinical scoring of autonomic tests. • Based on clinical autonomic scoring, patients with uncontrolled epilepsy had a higher degree of autonomic dysregulation.
Ferri et al. (43)	• Eleven children (5 males and 6 females) with a mean age ± SD of 11.5 years ± 3.65 • The control group consisted of 11 (5 males and 6 females) individuals aged (mean ± SD) 12.9 years ± 2.72	• All children had partial epilepsy and were treated with one or more ASMs. • Diagnosis was based on EEG and neuroimaging.	• Sleep EEG • Time and frequency domain HRV measurements	• The study showed that in the patients with epilepsy during sleep had lower time and frequency domain HRV values. • The sympathovagal balance (LF/HF) ratio was higher in patients with epilepsy especially during sleep when compared to the control group. • During REM sleep there can be altered autonomic patterns in children with partial epilepsy.
Yang et al. (44)	• Thirty children (21 males and 9 females) with a mean age ± SD of 6.0 years ± 1.3 • The control group consisted of 30 age and gender matched healthy individuals without a history of neurodevelopmental disorders.	• Of the 30 children, 22 also had neurodevelopmental disorders such as cerebral palsy and developmental delay. • The profile of seizures included 18 cases of GTC seizures, 10 with partial seizure and 2 with absence seizures.	ECG measurements and frequency domain HRV analysis.	• Mean age of seizure onset was 26.6 months and the mean length of seizure disorder was 4.6 years. • Children with epilepsy were noted to have an abnormal sympathovagal imbalance.

*ASMs, Anti-Seizure Medications; EDA, Electrodermal Activity; EEG, Electroencephalography; ECG, Electrocardiogram; GCS, Generalized Convulsive Seizures; GTC, Generalized Tonic Clonic; HF, High Frequency; HRV, Heart Rate Variability; LF, Low Frequency; MRI, Magnetic Resonance Imaging; PGES, Post-ictal Generalized Electroencephalographic Suppression; QTcD, QTc dispersion; RMSDD, Root Mean Square of Successive Differences; R-R, Inter-beat Interval; RSA, Respiratory Sinus Arrhythmia; SD, Standard Deviation; SDNN, Standard Deviation of all NN Intervals; SUDEP, Sudden Unexpected Death in Epilepsy*.

### Study Characteristics

The studies were expansive in terms of evaluating different aspects of autonomic dysregulation in children with epilepsy. This included assessment of heart rate variability (HRV) parameters before and after GTC seizure onset with and without Post-ictal Generalized Electroencephalographic Suppression (PGES) (28). Indices of HRV were also used to profile focal and generalized seizures in children (30). Another study explored HRV parameters in intractable epilepsy (32) or children with epilepsy during sleep (35). These studies were useful to see if patterns in the sympathovagal balance could be identified to assist in the development of potential prognostic markers for SUDEP. The developmental trajectory of PGES alongside the amplitude of electrodermal activity (EDA) across different age ranges was also explored (36). In some studies, specific aspects of HRV indices were assessed in children with refractory epilepsy and compared to those where the epilepsy was better controlled (37). Properties of the R-R interval during the pre-ictal period were also assessed (38). The relationship between PGES and peri-ictal tachycardia and hypoxemia in children with epilepsy was examined. This study was useful because the observations could also be correlated with SUDEP-7 inventory scores (29).

The interplay between different components of the autonomic nervous system (ANS) was also assessed in generalized sub-clinical and seizures of temporal origin (39). This aspect was further explored in intractable and well-controlled epilepsy (31). The autonomic characteristics of patients on ASMs and how this compares to patients without treatment were also investigated (40). Some other studies provided a broader overview of the sympathovagal profile in children with epilepsy (41–44).

### Thematic Analysis

**Figure 2:**
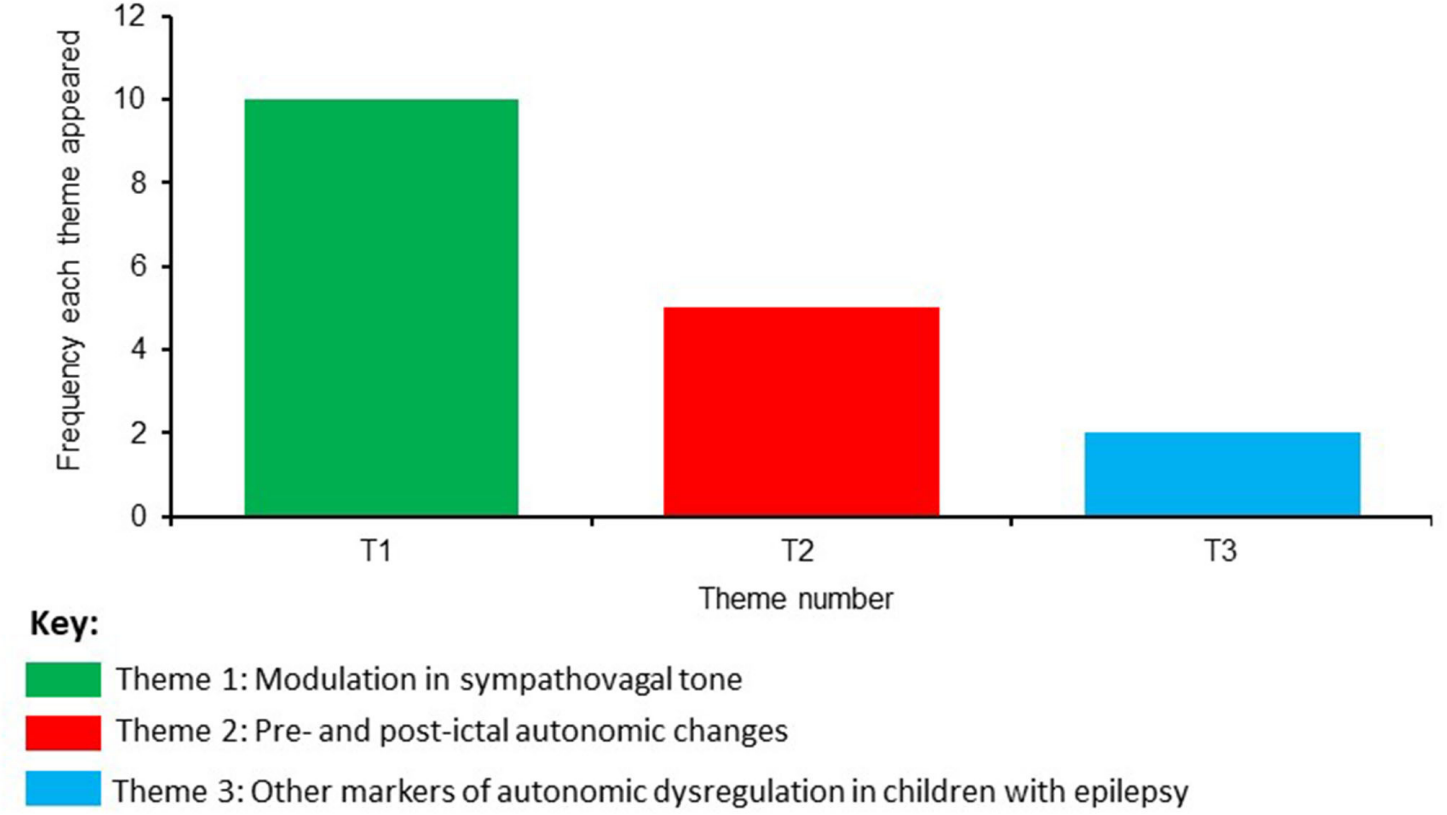
Frequency of identified themes.

Based on a consensus agreement between all the authors, three themes emerged from the eligible studies evaluating SUDEP in the pediatric population. The frequency of these themes is shown in Figure 2 and are named as:

Theme 1: Modulation in sympathovagal tone
Theme 2: Pre- and post-ictal autonomic changes
Theme 3: Other markers of autonomic dysregulation in children with epilepsy.

The most frequent theme that emerged was regarding the modulation in sympathovagal tone, followed by autonomic changes before and after seizure onset (pre- and post- ictal autonomic changes). The theme with the lowest frequency was related to other markers of autonomic dysregulation. The main results from these themes will be described in the next section:

#### Theme 1: Modulation in Sympathovagal Tone

Changes to the sympathovagal tone emerged from studies that investigated the autonomic characteristics of epilepsy in children to assess whether there is an underlying autonomic dysregulation. One important aspect that arose from this theme was the detection of sympathovagal changes before seizure onset and how this might alter based on seizure localization. For example, it was shown that shortly after focal seizure onset, there is tachycardia, decreased HRV, and increased sympathovagal imbalance as indicated by an increased Low Frequency/High Frequency (LF/HF) power (30). However, when assessing the autonomic phenotype of generalized seizures, it was found that children had tachycardia, decreased Root Mean Square of Successive Differences (RMSSD) and increased LF/HF power before seizure onset. These observations provide evidence for a differential diagnosis of seizure phenotype from the perspective of autonomic indices between focal and generalized seizures in children. In particular, it suggests that during the pre-ictal period, there is a vagal decline characterized by a sympathovagal shift toward the sympathetic component in children with epilepsy. From a clinical viewpoint, this is relevant because (I) peri-ictal reductions in vagal tone can increase cardiac dysfunction leading to short term changes in tachycardia and fibrillation (38, 45) and (II) a decrease in RMSSD has been shown to be associated with higher total scores on the SUDEP-7 risk inventory (9, 46).

The sympathovagal decline also seems to be a characteristic phenotype in children with refractory epilepsy. These children are also suggested to have lower time, frequency and non-linear domain HRV parameters when compared to age and gender-matched controls (32), and it was suggested that the decline in vagal tone is due to decreases in both the sympathetic and parasympathetic components of the ANS. The amplitude of this change was also demonstrated to increase at night and peaked in early morning (32). This aligns with other data in children with generalized epilepsy that show increased respiratory sinus arrhythmia and lower heart rate during sleep than control subjects (35). This study also suggested that an elevated parasympathetic tone is an autonomic characteristic that precedes the onset of seizures in children. Another study had also demonstrated decreased time and frequency domain HRV parameters during sleep in children with epilepsy (43).

Modulation in sympathovagal tone can also be useful in pinpointing features between generalized sub-clinical seizures to those that originate from the temporal lobe. In another study, generalized subclinical seizures were also shown to present with increased parasympathetic activity when compared to seizures originating from the temporal lobe, and could indicate an increased autonomic vulnerability in children with generalized sub-clinical seizures (39). When looking more specifically at epilepsy that is intractable and comparing it with control subjects, there is evidence to suggest a higher autonomic dysregulation in children with intractable epilepsy (31), which could be driven by a decrease in the parasympathetic component (41), and reduction in time domain HRV parameters (42). In summary, time-domain and HRV parameters are reduced in children with epilepsy, and those not on any ASMs showed a trend toward a more suppressed parasympathetic autonomic profile (40).

#### Theme 2: Pre- and Post-ictal Autonomic Changes

Following the theme concerning changes to the sympathovagal tone, the second most frequent theme that emerged was related to pre- and post-ictal autonomic changes. Post-ictal generalized EEG suppression (PGES) occurs after a seizure, and it has been suggested that PGES may be a reflection of brainstem shutdown and a failure of arousal mechanisms (28). This adds weight to the hypothesis that PGES could be a potential marker for SUDEP, especially in instances where its duration is longer than 50 s (47), however, others have indicated that the duration of PGES does not seem to be a risk factor for SUDEP (48, 49). Notwithstanding this inconsistency, in children with generalized seizures, pre-ictal autonomic parameters (LF and HF) were found to be higher in those children with Generalized Convulsive Seizures (GCS) and PGES than without PGES (28). This suggests that children with GCS and PGES together have a more disturbed autonomic dysregulation and potentially higher risk of SUDEP. Post-ictal RMSSD values were also higher in children with GCS and PGES. Higher post-ictal RMSDD in children with GCS with PGES could be a pre-cursor for events leading to SUDEP. There is limited data on the neurodevelopmental risk of SUDEP. Some evidence has shown that the duration of PGES is associated with age with adults having a longer duration of PGES than children and that sympathetic activity during the pre-ictal period correlates with the duration of PGES (36).

Assessment of the R-R interval can also be useful in detecting pre-ictal heart rate changes in temporal and frontal lobe seizures. In seizures of temporal or focal localization, the pattern of heart rate changes was shown to be different pre and post-seizure onset (38). However, this pattern was not found in children with generalized seizures. Generalized seizures in children are noticeable due to PGES (29). In this study, PGES was reported in about 16% of the seizures in 32% of children. The average duration of PGES was 35.1 ± 19.6 s, and in 10 children, the duration of PGES was ≥30 s. Peri-ictal tachycardia was the most frequent autonomic characteristic noted in about 40% of seizures. While there was no significant association between peri-ictal tachycardia and the duration of PGES, the presence of peri-ictal tachycardia did show a significant association with PGES (*P* = 0.019). Similarly, PGES was shown to be associated with peri-ictal hypoxemia (*P* = 0.005) and there was also a trend toward peri-ictal hypoxemia and the duration of PGES (*P* = 0.054). Children with PGES were also shown to have higher scores on the SUDEP-7 inventory (*P* = 0.007). When viewed together, the findings from this study demonstrate that (I) in children with PGES there is an association with the presence of peri-ictal tachycardia and hypoxemia and (II) following a generalized seizure, the occurrence of PGES could potentially increase the risk of SUDEP in these children.

#### Theme 3: Other Markers of Autonomic Dysregulation in Children With Epilepsy

This theme incorporated other potential markers of autonomic dysregulation in children with epilepsy. When GTC seizures were compared between adults and younger patients, children were shown to have higher EDA values (36). Since EDA reflects changes in sympathetic activation (50, 51), this finding suggests that when controlling for the length of PGES, children with GTC seizures have higher sympathetic activation than adults. When looking at cardiac parameters, children with refractory epilepsy were found to have prolonged QTc dispersion (QTcD) (37). This finding is important because a previous 2-year review of seizures in a pediatric unit showed ictal arrhythmias were present in 40% of patients (34) and might suggest that patients with epilepsy are more predisposed to increases in QTcD.

### Quality Appraisal of the Eligible Articles

Each of the 15 included articles in the study was assessed against 11 eligibility criteria (Table 2). The majority of the studies included children, but two had a mixed population (36, 39). None of the studies provided a formal sample size estimate to determine whether the studies were sufficiently powered; however, some studies did acknowledge this limitation. Studies also used a variety of methods ranging from EEG, HRV and specific tests for autonomic and cardiac function. While the studies do provide important information regarding the autonomic characteristics of HRV and its potential as a prognostic biomarker in SUDEP, none of the studies included data specifically on SUDEP in children, and only one study had correlated the findings related to PGES to the SUDEP-7 inventory (29). This finding is not unexpected as there is very limited data regarding SUDEP in children. While the topic of SUDEP has been discussed previously (5, 33), there is very little empirical data regarding pediatric SUDEP. In the present evidence synthesis, the quality appraisal suggests that none of the identified studies can provide direct, clinically meaningful inferences regarding SUDEP in children. Despite this limitation, the studies were valuable in providing information on autonomic characteristics that would be useful for managing risk in pediatric SUDEP. The SUDEP-7 inventory is a surrogate measure of SUDEP risk and includes two potential biomarkers of SUDEP risk—RMSSD (46) and PGES (29, 36), and the thematic analysis showed that RMSDD and PGES are factors that should be considered for managing risk in pediatric SUDEP. Furthermore, epilepsy can itself cause reductions in HRV (37) and respiratory sinus arrhythmia together with mean heart rate can identify children with epilepsy before the clinical signs become apparent (35).

**Table 2:** Quality assessment of reviewed studies on autonomic characteristics in children with SUDEP.

**Study**	Criteria*										
	**1. Was the sample characteristic of the specific population?**	**2. Were patients recruited in an appropriate way?**	**3. Was the sample size sufficient to power the study?**	**4. Were the study participants described in detail and fosters comparison with other relevant studies?**	**5. Was the data analysis undertaken with adequate description of the identified sample?**	**6. Were objective and standard criteria used for the measurements?**	**7. Were the assessment and measurement methods used reliably?**	**8. Were the statistical analyses used appropriate?**	**9. Were relevant confounding factors described and accounted for?**	**10. If sub-populations were identified, were they done according to objective criteria?**	**11. Was there a conflict of interest?**
Okanari et al. (28)	No—children with PGES and healthy age matched controls were included but cohort did not specifically examine SUDEP	N/A—review of retrospective data collection	Unclear (the authors mention that the study was performed in a single tertiary care center and the cohort did not include information from a population based sample)	Yes	Yes	Yes, EEG and HRV measurements were used.	Yes	Yes, to compare autonomic characteristics with and without PGES	Yes, sleep stages that could influence HRV was mentioned	N/A	No
Pernice et al. (30)	No—cohort did not specifically assess SUDEP	Yes	Unclear—no information about sample size estimates were provided	Yes	Yes	Yes	Yes	Yes, nine indices of HRV were analyzed.	Yes	N/A	Unclear—no conflict of interest statement was provided
Yang et al. (32)	No—the study did not specifically include SUDEP	Yes—although the study included a mixed age range from 6 to 38 years	Unclear—although it was mentioned more sampling and accurate data would be needed for further SUDEP studies	Yes	Yes	Yes, ECG data was analyzed using Kubios software and cosinor fit method was used to assess the circadian HRV rhythm	Yes	Yes, time, frequency and non-linear domain indices were analyzed	Yes, the different phenotypes of epilepsy in the patient population was mentioned along with the ASM of patients	N/A	No
Sivakumar et al. (35)	No—data from SUDEP was not specifically assessed	N/A—retrospective data collection from a single pediatric epilepsy unit	Unclear—the need for further subjects was indicated including those with focal epilepsy	Yes	Yes	Yes	Yes	Yes, analyses of ECG and EEG data	Yes	N/A	No
Sarkis et al. (36)	No—although the paper stated implications for SUDEP, the study cohort did not include data from SUDEP	Yes—study population was mixed (13 were adult patients aged 18–67 years)	No—the authors mention that the study was limited due to the small sample size (only subjects with GTC seizures were analyzed)	Yes	Yes	Yes	Yes	Yes, EEG, HRV and EDA analyses	Yes, age and seizure capture were mentioned	N/A	Yes, a disclosure statement for the authors was provided
Kolsal et al. (37)	No—the cohort did not assess SUDEP	Yes	Unclear—no indication of sample size estimates was mentioned	Yes	Yes	Yes	Yes	Yes, HRV analyses	Yes	Yes, alongside refractory epilepsy a well-controlled epilepsy group was also included	No
Jansen et al. (38)	No—SUDEP was not assessed	Yes	Unclear, although the limitations of the small sample were considered when comparing the difference between temporal lobe (mesial and lateral) seizures	Yes	Yes	Yes	Yes	Yes, EEG and ECG analyses	Yes	N/A	Unclear as no conflict of interest statement was provided
Moseley et al. (29)	No—children with PGES had higher SUDEP-7 inventory score but the study cohort did not include information specifically related to SUDEP	Yes	Unclear—the study analyses took account the small sample size and fewer patients with PGES	Yes	Yes	Yes	Yes, including a surrogate measure of SUDEP (SUDEP-7 inventory)	Yes	Yes, the authors described this in detail	Yes	Unclear as not conflict of interest statement was provided
Brotherstone and McLellan (39)	No—although a SUDEP mechanism was proposed the study did not formally assess SUDEP	Yes—study was a mixed population (six patients were adults)	N/A—study was a pilot study	Yes	Yes	Yes	Yes, the use of Neuroscope and BioSignal HRV	Yes	Yes	N/A	No
Mukherjee et al. (31)	No—sample did not formally assess SUDEP	Yes	No—the study was not sufficiently powered to explore gender and age	Yes	Yes	Yes, a range of tests for autonomic function were performed	Yes	Yes and the small sample size was factored into the analyses	Yes	N/A	Unclear—as no statement was given
Hallioglu et al. (40)	No—the cohort did not assess SUDEP	Yes	Unclear—although it was mentioned that the sample size was too small when groups were split based on their ASMs	Yes	Yes	Yes	Yes, time and frequency domain measure of HRV	Yes	Yes	Yes, were divided into sub-groups based on ASMs	Unclear—no statement was provided
Harnod et al. (41)	No—no assessment of subjects with SUDEP	Yes	Unclear—no sample size statement provided	Yes	Yes	Yes	Yes, frequency domain measurements of HRV	Yes	Yes—patient characteristics (such as the exclusion of those with partial or controlled seizures)	N/A	Unclear—no statement provided
El-Sayed et al. (42)	No—cohorts did not include information on SUDEP	Yes	Unclear—although the small number of patients was acknowledged in the study.	Yes	Yes	Yes, five tests for cardiac autonomic function	Yes	Yes	Yes	N/A	Unclear—no statement was provided
Ferri et al. (43)	No—study did not include information on SUDEP	Yes	Unclear—however, the size of the study group did limit the comparisons between right vs. left side EEG abnormalities	Yes	Yes	Yes	Yes	Yes	Yes—possible influence of ASMs on HRV was indicated	N/A	Unclear—not statement was provided
Yang et al. (44)	No—no data concerning SUDEP was included in the study	Yes	Unclear -although the authors do acknowledge the small sample size that limits the generalizability of the study findings	Yes	Yes	Yes	Yes—frequency domain HRV analysis	Yes	Yes—differences in hemisphere effects were mentioned	N/A	Unclear—no information was provided

*ASMs, Anti-Seizure Medications; ECG, Electrocardiogram; EDA, Electrodermal Activity; EEG, Electroencephalograph; GTC, Generalized Tonic Clonic; HRV, Heart Rate Variability; PGES, Post-ictal Generalized Electroencephalographic Suppression; N/A, Not Applicable; SUDEP, Sudden Unexpected Death in Epilepsy*.

* *Ratings were: Yes (fully meeting the criterion), No (not meeting the criterion), Unclear (unclear to whether the criterion was met), and N/A (criterion was not applicable) as previously described (20)*.

In summary, the quality appraisal shows that while no direct clinical comparison can be made from the information provided in the articles to pediatric SUDEP, the thematic analysis does suggest that the autonomic characteristics of the studies would be useful for managing risk is pediatric epilepsy. This aligns with a recent systematic review of SUDEP in children that suggested even though the data relating to the causes of pediatric SUDEP is limited, the best way to reduce the risk of SUDEP in children is to optimize the management of epilepsy (33). This principle would be especially relevant in patient groups who are particularly more vulnerable to seizures such as those with RTT (14, 17, 52).

## Discussion

The findings from the systematic review showed that children with epilepsy have (I) an altered sympathovagal tone, (II) have discernible pre- and post-ictal autonomic changes, and (III) have suggestive biomarkers of autonomic dysregulation namely changes in EDA and QTcD. While none of the studies provides direct information relating to pediatric SUDEP, there is some indication that pre- and post-ictal autonomic features could be predisposing risk factors for SUDEP. In line with this view, we wanted to extrapolate the current findings to see if it would provide useful information concerning the management of SUDEP in patients with RTT. We are cognisant of the fact that it would be difficult to predict the onset of epileptiform events in patients with RTT without formal ambulatory or video EEG assessment. This is due to the nature of non-epileptic vacant spells that occur in patients with RTT (52). The characteristic stereotypical movements such as hand movements and dystonia can also make identification of epilepsy in Rett patients more difficult (53). Moreover, another study showed that non-epileptic episodes can also consist of laughing, pupillary dilation and breathing dysregulation (14). This also aligns with the finding that even though seizures are common in RTT, many suspected seizures do not show characteristic epileptiform events on video EEG monitoring (54).

In Rett patients, it is not understood why some seizures are followed by suppression of electrical brain activity on the EEG (PGES). It is possible in RTT, that this could be down to a random event; however, some others have proposed that the events leading to a seizure might also be predictable (55) and be dependent on autonomic mechanisms. In this context, we wanted to address the following questions:

1. What do the autonomic characteristics of SUDEP in children tell us about possible SUDEP in patients with RTT?

2. Can autonomic indices be used to develop biomarkers to identify clinical risk factors of SUDEP in RTT?

### What Do the Autonomic Characteristics of SUDEP in Children Tell Us About Possible SUDEP in Patients With RTT?

At present, information on the autonomic events that could precipitate SUDEP in patients with RTT is unknown. We have previously alluded that patients with RTT could be more vulnerable to changes that could lead to SUDEP (20), however, in RTT it is unclear whether the risk of SUDEP changes across the age range. In RTT, the patterns of seizures come and go, and can be sporadic. If indeed focal epilepsy appears to be more frequent than generalized epilepsy in RTT (52), then it might be possible to detect changes in sympathovagal tone and distinguish focal from generalized seizures as described previously (30).

In the British Isle survey of 137 RTT patients, of the 89 subjects that responded the prevalence of epilepsy was 67 and 62% of patients had GTCs (52). Epilepsy severity data from 736 patients from the Rett Networked Database showed that 55% had seizures classified as grade 1, i.e., well-controlled, and about 32% were judged to be of grade 2 (uncontrolled seizures). GTCs were present in about 46% of patients (17) while in the Natural History Study, ~46% of patients had focal onset seizures while generalized seizures were noted in 47% of patients (14). These findings indicate that GTC seizures are quite common in patients with RTT, however, there is no empirical evidence to determine what the likelihood of PGES occurring following a generalized convulsive seizure (GCS) in RTT. In the EEG, cessation of background activity is indicative of PGES, and it is during this post-ictal state that patients are at most risk to abnormal cardiorespiratory events (56). A recent evidence synthesis has indicated that in RTT there is a diffuse reduction in the background EEG activity (57) but whether this reduction in background activity meets the threshold of PGES in Rett patients is unknown. The findings from the current review show that (I) in children with epilepsy, PGES is not an uncommon finding, (II) PGES is associated with peri-ictal tachycardia and hypoxemia, (III) pre-ictal LF and HF are higher in children with GCS and PGES, and (IV) post-ictal RMSSD was elevated in children with GCS and PGES compared to those with GCS alone. In children, PGES is also associated with higher scores on the SUDEP-7 inventory.

These findings have implications for patients with RTT because given the underlying brainstem vulnerability and electrical instability of the cardiovascular system, there is a risk of PGES occurring following generalized seizures in this patient group. Whether PGES could increase the risk of SUDEP is a matter of debate (47–49). However, it is probable that patients with RTT could be more vulnerable due to the underlying autonomic cardiorespiratory dysfunction alongside generalized seizures. In the MORTEMUS study, SUDEP cases had a characteristic pattern of respiratory distress, PGES, and then apnoea followed by bradycardia (56). In RTT, abnormal EEG activity can also occur without obvious clinical seizures (52) and this is important because both convulsive and non-convulsive seizures tend to change cardiorespiratory function (58). Even though, post-ictal tachycardia and hyperventilation took longer to return to baseline in convulsive seizures (58), in RTT patients the underlying autonomic dysregulation may further exacerbate the post-ictal tachycardia and hyperventilation, even in those patients with no overt signs of clinical seizures.

Children with GCS and PGES have higher SUDEP-7 inventory scores (29). There is also a correlation between the duration of PGES and age (36). This suggests that adults would be at higher risk of SUDEP; however, it is unknown if this would also be the same for the RTT population. We have surmised that the ANS in children with RTT could be particularly more sensitive to autonomic changes (59). Children with PGES do have an abnormal sympathovagal tone characterized by a greater post-ictal sympathetic activation. During the pre-ictal period of children with GCS and PGES, the time domain parameters of LF and HF are raised (28). These HRV indices reflect changes in parasympathetic and sympathetic vagal tone, and in the most vulnerable RTT patients, it would be prudent to monitor LF and HF changes to detect early signs of abnormal vagal tone, which could help to manage the risk in these patients.

### Can Autonomic Indices Be Used to Develop Biomarkers to Identify Clinical Risk Factors of SUDEP in RTT?

It is clear from the findings that in children with epilepsy and PGES, there is a modulation in sympathovagal tone (28). The raised LF and HF power before seizure onset suggest increases in sympathetic and parasympathetic tone. Interestingly, the RMSDD, which is a measure of parasympathetic tone, was sustained during the post-ictal period in children with PGES. This sustained increase in RMSDD points toward an increased parasympathetic modulation. Metrics of HRV can also provide information on the seizure phenotype. Children with generalized seizures show a trend of lower RMSSD but higher LF and LF/HF before the seizure (30). In RTT patients, there is some evidence of lower RMS (60). Changes in EDA could also provide useful information on seizures since when compared to adults, children with epilepsy were found to have a higher EDA response (36). We have previously shown that the EDA is disordered in RTT patients (61, 62). In our recent evidence synthesis (59), we proposed that EDA could also be useful in monitoring the physical health of the patient, and we can now extend its use to provide valuable information on the sympathetic response in RTT patients who are more prone to frequent seizures.

**Figure 3:**
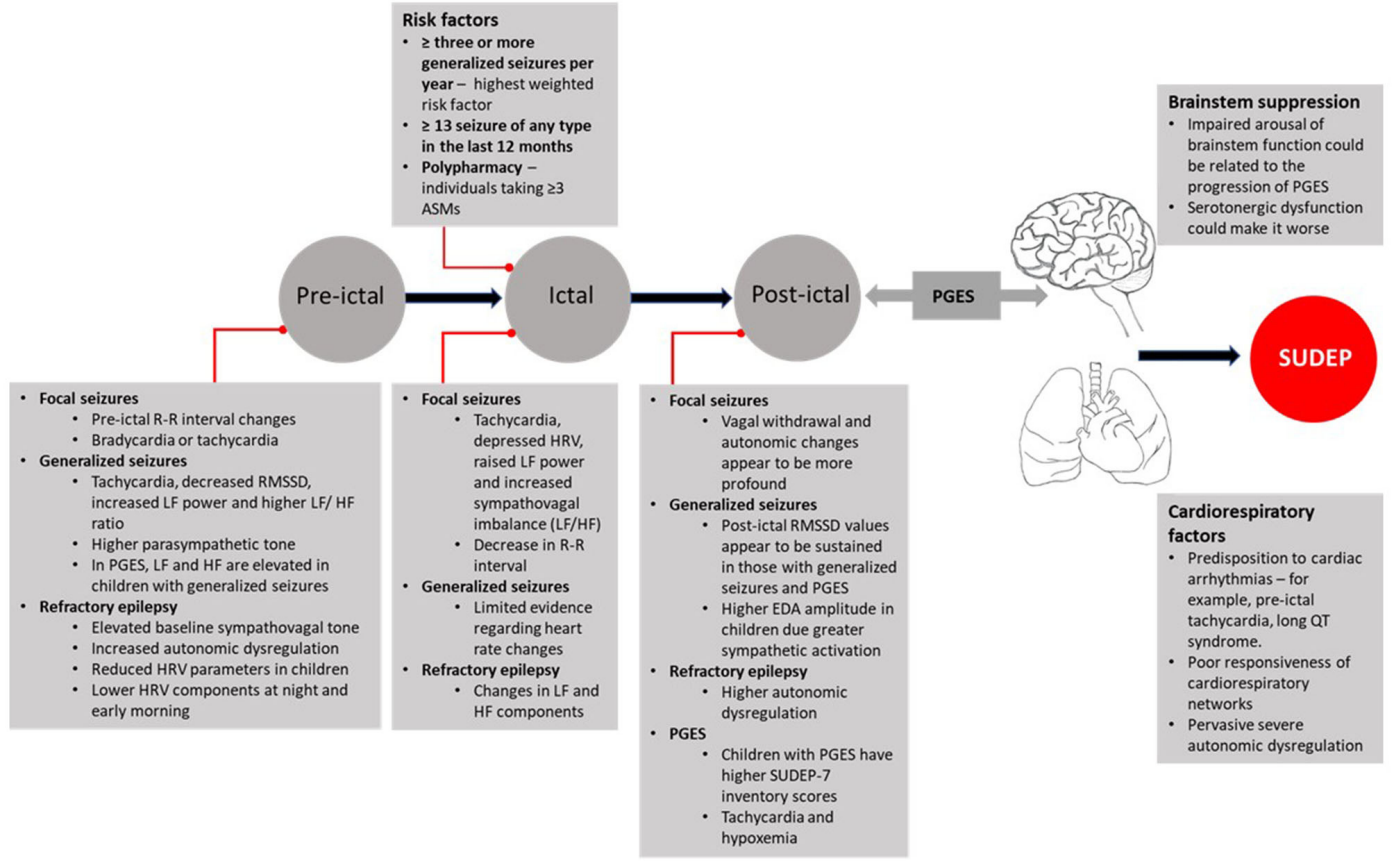
Autonomic characteristics of pre-ictal, ictal, post-ictal states, and propensity to SUDEP. ASMs, Anti-Seizure Medications; EDA, Electrodermal Activity; HF, High Frequency; HRV, Heart Rate Variability; LF, Low Frequency; PGES, Post-ictal Generalized Electroencephalographic Suppression; QT, Q and T waves on ECG [electrocardiogram]; RMSSD, Root Mean Square of Successive Differences; R-R, Inter-Beat Interval; SUDEP-7, Sudden Unexpected Death in Epilepsy Risk Inventory.

While the incidence of SUDEP in patients with RTT is unclear what the current evidence synthesis tells is that given the changes in the sympathovagal tone, Rett individuals could be more susceptible to autonomic changes before and during a seizure. It is clear from the evidence in children with epilepsy that an autonomic derangement occurs leading to fluctuations in sympathetic to parasympathetic shifts and vice versa. It is also apparent that there are characteristic autonomic changes in the pre-ictal period that extend into the post-ictal period. Figure 3 presents a summary of these changes. In RTT, it should also be borne in mind that the event of SUDEP will also depend on other measures of susceptibility such as the associated post-ictal cardio-respiratory state, and hence the events leading to SUDEP would be very difficult to predict. Despite these limitations, it would be useful to monitor SUDEP-7 ratings, HRV metrics and EDA in RTT patients to see we if can identify patterns in the autonomic dysregulation before and after seizure onset. While we would not be able to determine why some of the seizures in Rett patients progress to SUDEP, this strategy might allow the risk stratification of the most vulnerable patients especially in instances where the presence of clinical seizures is not obvious and epileptiform activity by EEG monitoring is not readily available.

## Conclusion

This is the first study that had conducted a quality appraisal and thematic analysis on the autonomic characteristics of SUDEP in children. While direct evidence regarding studies on pediatric SUDEP is low, the information learned from this systematic review does allow further understanding of the autonomic profile in pediatric epilepsy and the events that could lead to SUDEP. This information is useful for optimizing the management of epilepsy in patients with RTT because it provides evidence on how important it is to obtain the best seizure control in this patient group.

Pediatric SUDEP is heterogeneous and likely to be driven by a range of factors. Brainstem suppression could be a common mechanism (4), however, it is less certain how events in the early pre-ictal phase develop into a more severe post-ictal phase and in some instances to death. Serotonergic dysfunction could play a role. Recently in a prospective multicenter study of SUDEP in 49 patients, it was shown that higher levels of post-ictal serum 5-HT were associated with reduced seizure related breathing dysregulation and this increase might protect against the deleterious changes leading to SUDEP (63).

The present evidence synthesis suggests that in children with epilepsy, the sympathovagal balance is impaired and there are also subtle changes in autonomic characteristics pre- and post- seizure onset. In patients with RTT, the epilepsy is likely to cause fluctuations in HRV and EDA because of the dysregulation in the central autonomic network. Following the onset of a seizure, in typical circumstances, there would be a decrease in vagal tone with a concomitant increase in heart rate (39), however the underlying autonomic dysregulation in RTT would lead to fluctuations in this vagal tone, and this could predispose patients to sympathetic storming, which would have an impact on the ascending control of brainstem functions. In Rett patients there are serotonergic abnormalities (20) and serotonergic agents have been shown to reduce SUDEP in animal models (64). As post-ictal cardiorespiratory states are less agile in RTT, one possible option would be to consider serotonergic agents especially in Rett patients whose seizures are poorly controlled to see if the recovery time post-seizure can be reduced. Robust clinical trials would be needed to test this hypothesis specially in the RTT patient population.

Previous data suggest that GTC seizures are the most common seizure phenotype in SUDEP (21, 22, 65). In RTT the frequency of generalized seizures range from 62 to 46% (14, 17, 52); however further work would be needed to identify the most common seizure phenotype that leads to SUDEP in RTT and also why some seizures in RTT terminate while some might eventually lead to SUDEP. Desynchronization in seizure mechanisms could result in a summation of events that cause brainstem shutdown in SUDEP (66) and the current findings show that after seizure onset, RMSDD remains sustained in children with generalized convulsive seizures and PGES (28) suggesting a more severe autonomic dysregulation post-seizure. Increases in EDA have also been documented during the post-ictal period (36, 67) and this increase could provide critical information alongside HRV measurements in Rett patients. Measurement of EDA using a non-invasive wearable sensor offers an alternative way to monitor seizure outcomes in RTT patients and could optimize the management of seizures in this vulnerable patient group. This would also help to support EEG findings for the diagnosis of epilepsy, as in RTT, the EEGs can be abnormal even when there are no seizures (14). Typically, EEGs would need to be performed during an ‘event', but because these EEGs are usually performed in a clinical setting, infrequent seizures could be missed. In this scenario, the use of wearable sensors to detect subtle pre-ictal changes in EDA and HRV (alongside EEG monitoring) would be beneficial in this patient group.

In summary, the limitation in identifying direct inferences regarding SUDEP underscores the need for further research on this topic in the pediatric population. Even though the mechanism leading to SUDEP are likely to be complex and involve post-ictal cardiorespiratory mechanisms, in vulnerable populations where there is already an autonomic dysregulation, monitoring the autonomic characteristics of EDA and HRV pre- and post-seizure onset using non-invasive wearable sensors would be beneficial for managing the SUDEP risk.

## Limitations

The level of evidence regarding the autonomic characteristics of pediatric SUDEP is low, as there has been no specific study of SUDEP in RTT, and the extrapolation of information to the broader RTT community should be placed in this context. While the review shows that autonomic characteristics would be useful in managing epilepsy risk in RTT, given the probabilistic nature of SUDEP, the findings from the present study should be interpreted with caution because they do not show that monitoring these autonomic characteristics would prevent SUDEP. We have also proposed that autonomic dysregulation follows a non-linear trajectory (20) and given that the time course of epilepsy in RTT fluctuates across the lifespan (14, 17, 52), HRV and EDA measurements would not be uniform, and without higher-order analytics it would be difficult to predict autonomic features of seizure onset across different patient sub-groups. The patients in the studies evaluated were also on different ASMs, and we cannot exclude the role ASMs would have had on the trajectory of autonomic dysregulation and whether these medications influence HRV.

## Data Availability Statement

The datasets generated for this study are available on request to the corresponding author.

## Author Contributions

JS developed the idea of the study and wrote it. The systematic searches of the databases were undertaken by JS and EL in an independent and blinded manner. EL reviewed the thematic analysis and quality appraisal of the articles. Both EL and PS reviewed the scientific content of the draft and final versions. All authors have read and approved the final manuscript.

## Conflict of Interest

PS was a Principal Investigator (PI) on the Sarizotan (Protocol Number Sarizotan/001/II/2015; ClinicalTrials.gov Identifier: NCT02790034) and is currently the PI for the Anavex Life Sciences Corp. (Protocol Number: ANAVEX2-73-RS-002) clinical trial in Rett Syndrome (RTT). PS is the co-inventor of the HealthTracker^TM^ and is the Chief Executive Officer and shareholder in HealthTracker^TM^. JS was a Trial Research Methodologist on the Sarizotan Clinical Trial (Protocol Number Sarizotan/001/II/2015; ClinicalTrials.gov Identifier: NCT02790034) in patients with RTT and is a Research Manager on the Anavex Life Sciences Corp. (Protocol Number: ANAVEX2-73-RS-002) clinical trial for RTT. JS is also an advisor for Reverse Rett. The remaining author declares that the research was conducted in the absence of any commercial or financial relationships that could be construed as a potential conflict of interest.

## Abbreviations

ASMs: Anti-Seizure Medications
ECG: Electrocardiogram
EDA: Electrodermal Activity
EEG: Electroencephalograph
GTC: Generalized Tonic Clonic
HF: High Frequency
HRV: Heart Rate Variability
LF: Low Frequency
PGES: Post-ictal Generalized Electroencephalographic Suppression
QT: Q and T waves on ECG
RMSSD: Root Mean Square of Successive Differences
RTT: Rett Syndrome
R-R: Inter-Beat Interval
SIDS: Sudden Infant Death Syndrome
SUDC: Sudden Unexplained Death in Childhood
SUDEP: Sudden Unexpected Death in Epilepsy
SUDEP-7: Sudden Unexpected Death in Epilepsy Risk Inventory.
